# A Zn-Ca-Based Metallic Glass Composite Material for Rapid Degradation of Azo Dyes

**DOI:** 10.3390/ma17133356

**Published:** 2024-07-07

**Authors:** Gaojiong Wang, Xin Wang, Wei Yang, Lichen Zhao, Yumin Qi

**Affiliations:** Hebei Key Laboratory of New Functional Materials, School of Material Science and Engineering, Hebei University of Technology, No. 5340, Xiping Road, Tianjin 300401, China

**Keywords:** metallic glass composite, zinc–calcium, in situ composite, azo dye degradation

## Abstract

The catalytic capabilities of metals in degrading azo dyes have garnered extensive interest; however, selecting highly efficient metals remains a significant challenge. We have developed a Zn-Ca-based metallic glass composite which shows outstanding degradation efficiency for Direct Blue 6. This alloy comprises a Zn_2_Ca crystalline phase and an amorphous matrix, allowing for the degradation of azo dyes within minutes in a wide temperature range of 0–60 °C. Kinetic calculations reveal an exceptionally low activation energy of 8.99 kJ/mol. The rapid degradation is attributed to the active element Ca and the unique amorphous structure of the matrix, which not only facilitates abundant redox conditions but also minimizes the hydrolysis of the active element. The newly developed metallic glass composite exhibits a notably higher azo dye degradation rate compared to those of general metallic glasses, offering a new avenue for the rapid degradation of azo dyes. This paper holds significant importance for the development of novel azo dye wastewater treatment agents.

## 1. Introduction

The development of the modern printing and dyeing industry has resulted in significant emissions of azo dye wastewater, gravely threatening water resources and the safety of the biosphere [1]. Azo dye wastewater boasts a high chemical oxygen demand (COD), typically ranging from 1200 to 2000 mg/L [2]. Upon oxidative decomposition, it depletes considerable amounts of dissolved oxygen in the water, fostering extensive growth of anaerobic bacteria, which ultimately leads to degradation of the aquatic environment and the development of unpleasant odors. Furthermore, azo dyes possess biological toxicity. Under specific conditions, they can decompose into various carcinogenic aromatic amines, and some contain heavy metal elements that pose severe risks to human health [3]. Consequently, it is imperative to subject azo dye wastewater to harmless treatment prior to discharge.

Oxidation–reduction reaction is one of the most useful theories for understanding the degradation of azo dyes, which mainly relies on additives or degradation materials. Zero-valent iron (ZVI), the most typical degradation material, acts as a reducing agent to directly react with azo dyes [4]. The typical oxidant ozone (O_3_) shows strong oxidizing properties with an oxidation potential in water reaching up to 2.07 V, which can directly oxidize the unsaturated bonds of azo dyes [5]. Moreover, the Fenton oxidation process harnesses the strong oxidizing properties of the hydroxyl radicals (•OH) formed by the reaction between ferrous ions (Fe^2+^) and hydrogen peroxide (H_2_O_2_) to degrade azo dyes [6]. In 2010, Zhang et al. reported that Fe-based metallic glass (MG) ribbons can efficiently degrade the azo dye Direct Blue 2B, showcasing higher catalytic activity and stability than ZVI [7]. When reacting with H_2_O_2_ in acidic environments, Fe-based MG alloys produced •OH with an oxidation potential of up to 2.80 V, making them efficient oxidants for the degradation of azo dyes [8]. Deng et al. employed the Cu_55_Zr_45_ MG alloy as an electrode, achieving rapid degradation of azo dyes through the electrocatalytic generation of •OH and anionic superoxide radicals (•O^2−^) [9].

Another viewpoint for understanding the mechanism of metals in degrading azo dyes is the galvanic cell reaction, with enhancing the density of the reaction sites identified as the most effective strategy. For instance, Chen et al. dealloyed Mg_73_Zn_22_Ca_5_ MG ribbons with citric acid, creating a Zn/ZnO porous framework that provided more reaction sites, significantly enhancing the degradation efficiency for azo dyes [10]. Zhang et al. fabricated Fe-based MG alloy porous scaffolds via selective laser melting, which were then electrochemically dealloyed to form nanocrystalline/MG phase galvanic cells, to enhance the catalytic performance by improving the electron transfer efficiency [11]. Jin et al. developed Mg-Zn-Ca-Sr metallic glass composites (MGCs), pointing out that the galvanic cell reaction between the crystalline Mg phase and the MG matrix is the primary factor in the rapid degradation of azo dyes [12].

The role of different metals in the degradation of azo dyes is complex. Wang et al. studied the degradation of azo dyes using a Al-Ni-Y MG alloy and found that the redox activity dominates in acidic environments, while under alkaline conditions, adsorption from corrosion product hydroxides is the main reason for organic compounds being removed [13]. Chen et al. investigated the effect of NaCl addition on the degradation performance of a typical Mg-Zn-Ca MG alloy, indicating that the photocatalytic degradation of zinc oxide contributes to the degradation of azo dyes [14]. In this study, we developed a zinc alloy with a high calcium content and found that the active element calcium plays an important role in the degradation of azo dyes.

## 2. Materials and Methods

A precision balance (accuracy 0.1 mg, Mettler, AB204-S, Columbus, OH, USA) was employed to weigh the master alloy with a nominal composition of Zn_45_Mg_11_Ca_40_Sr_4_, using Zn, Mg, Ca, and Sr with a purity of 99.99% as the raw materials. The master alloy was fabricated by vacuum induction heating under an Ar atmosphere. The master alloy ingots were re-melted and injected into a rotating copper roller at a speed of 2500 rpm in a spinning furnace (MTINST, SDM-0.02, Shanghai, China) to produce ribbons with a width of 3–6 mm and a length of over 10 cm. The cooled ribbons were ground into powder and sieved using 100–200-mesh screens. A metallographic microscope (ZEISS, Axio Imager M2m, DEU, Dublin, Germany) was used to take a picture of the powder, and the particle size of powder was statistically analyzed by using Image Pro Plus 6.0 software.

Commercial azo dye Direct Blue 6 powder (DB6, C_32_H_20_N_6_Na_4_O_14_S_4_, CHN) was mixed with deionized water at a ratio of 0.2 g/L to prepare the azo dye simulated solution. To investigate the azo dye degradation property, 0.2 g of Zn_45_Mg_11_Ca_40_Sr_4_ powder was put into 50 mL of azo dye solution, and the mixture was put into the water bath at different temperatures (0, 20, 40, 60 °C) and stirred by magnetic force. After stirring it for a certain time, about 0.3 mL of the treated solution was extracted with a syringe and filtered through the membrane filter (nylon). A UV–visible spectrophotometer (Yuanxi UV-6000, Shanghai, China) was used to test the absorbance of different solutions, with a testing wavelength range of 400–800 nm, a scanning speed interval of 1 nm, and a scanning accuracy of 10°. 

An X-ray diffractometer (XRD, Rigaku SmartLab, Cu-Kα radiation, Tokyo, Japan) was used to examine the phase composition of the powder samples with a scanning angle range of 10–90° and a scanning speed of 6°/min. A differential scanning calorimeter (DSC, Setaram Setline, Castelginest, France) was used to measure the thermal stability and glass transition characteristics of the samples at a heating rate of 20 K/min with N_2_ protection. A scanning electron microscope (SEM, HITACHI S-4800, Tokyo, Japan) was used to observe the surface micromorphology of the powder after the azo dye degradation experiments. X-ray photoelectron spectroscopy (XPS, Thermo K-Alpha, Al-Kα radiation, Norristown, PA, USA) was used to analyze the electronic states of various elements on the surface of samples after azo dye degradation.

## 3. Results and Discussion

**Figure 1:**
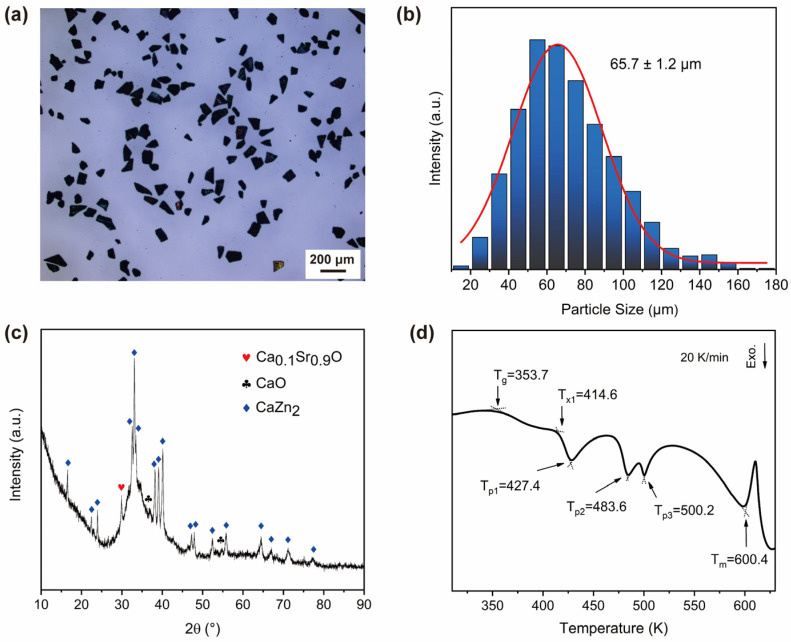
(**a**) Metallographic microscope photo of powder sample; (**b**) particle size statistic histogram; (**c**) XRD pattern; (**d**) DSC curve.

Figure 1 shows the macroscopic characterization results for the prepared powder sample to identify the phase composition. After manual grinding, the as-spun ribbons turn into small particles without irregular shapes (Figure 1a). Using Image Pro Plus software to analyze the particle size, a histogram of the particle size distribution was obtained, as shown in Figure 1b, indicating a particle size range of approximately 20 μm to 180 μm, with an average size of 65.7 μm. The XRD test result shown in Figure 1c indicates the presence of diffuse scattering in the pattern between 30° and 40°, suggesting the presence of a certain amount of the glassy phase. In addition, the XRD pattern shows sharp diffraction peaks corresponding to the crystalline phases CaZn_2_, Ca_0.1_Sr_0.9_O, and CaO, with weaker oxide peaks indicating low content. Oxides may originate from O impurities in the Ca and Sr raw materials, as they have a strong chemical affinity with oxygen and are easily oxidized during storage and transportation. The DSC curve in Figure 1d indicates that the powder sample undergoes a significant glass transition and a polycrystalline transition during continuous heating, with a glass transition temperature T_g_ of 353.7 K and an onset crystallization temperature T_x1_ of 414.6 K. This confirms the presence of a large amount of the glassy phase in the sample and validates the results in Figure 1c. In brief, this indicates that the prepared powder sample is a composite material consisting of an MG phase and a CaZn_2_ crystalline phase.

**Figure 2:**
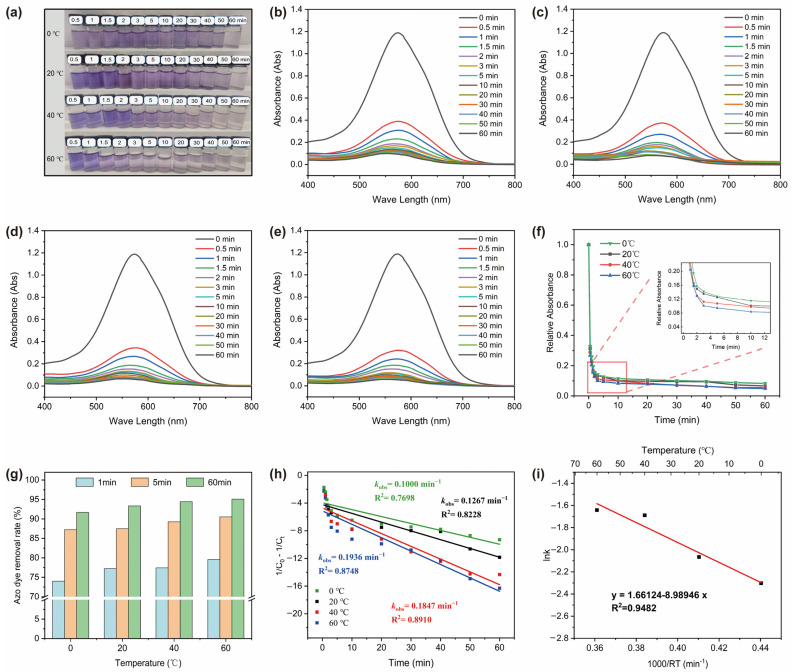
(**a**) Optical photos of residual solutions degraded for different times at different temperatures; (**b**–**e**) absorption spectra of residual solutions degraded for different times at 0, 20, 40, and 60 °C, respectively; (**f**) relative absorbance vs. degradation time curves at different temperatures; (**g**) azo dye removal rate; (**h**) degradation reaction kinetics curves at different temperatures, fitted according to second-order reaction kinetics equations; (**i**) Arrhenius equation fitting diagram.

Figure 2 shows the azo dye degradation properties of the Zn_45_Mg_11_Ca_40_Sr_4_ alloy powder based on DB6 solution at different temperatures. Based on the chromaticity of the residual solution after degradation for different times, the newly designed alloy powder can effectively decolorize DB6 solution at 0–60 °C. The higher the temperature of the degradation reaction, the faster the decolorization rate of the dye solution. Figure 2b–e show the UV–visible absorption spectra of the residual DB6 solutions. It can be seen that at 0.5 min, the absorption peak intensity of the residual solutions at all temperatures decreased by about 2/3, indicating that ~70% of the DB6 molecules were removed from the solution. We take the peak absorbance of each curve and normalize it based on the peak of the undegraded solution (relative absorbance = residual solution absorbance/undegraded solution absorbance) to obtain the azo dye degradation rate curve of the powder at different temperatures, as shown in Figure 2f (the inset is a local enlarged image of 2–10 min). This indicates that the reaction temperature has a certain effect on the degradation efficiency, and higher temperatures make the degradation reaction proceed faster. 

Figure 2g further presents the quantitative analysis results at degradation times of 1, 5, and 60 min. It can be seen that the degradation rate of the azo dyes is higher at higher temperatures with the same reaction time. It is worth noting that at room temperature (20 °C), the alloy powder degraded over 77% of the DB6 within 1 min, and the degradation rate of the azo dyes exceeded 93% at 60 min. It is demonstrated that the newly developed MGC composite has an excellent ability to rapidly degrade azo dye wastewater. In addition, according to the classical second-order reaction kinetics equation ($\frac{1}{C_{0}}-\frac{1}{C_{t}}=-k\times t$), the degradation curve was fitted, as shown in Figure 2h. The slope of the fitted equation is the reaction rate constant k_obs_, and the higher the temperature, the greater the k_obs_ value. This indicates that the degradation of azo dyes by alloy powder is a thermally activated process. By fitting the k_obs_ values of the four temperatures using Arrhenius equation ($lnk=-\frac{E_{a}}{RT}+lnA$), a reaction activation energy E_a_ = 8.99 kJ/mol was obtained (Figure 2g). This value is obviously lower than that of Fe-, Mg-, and Co-based amorphous alloys (15–80 kJ/mol) [15,16,17,18], indicating that their degradation reactions are easier to carry out.

**Figure 3:**
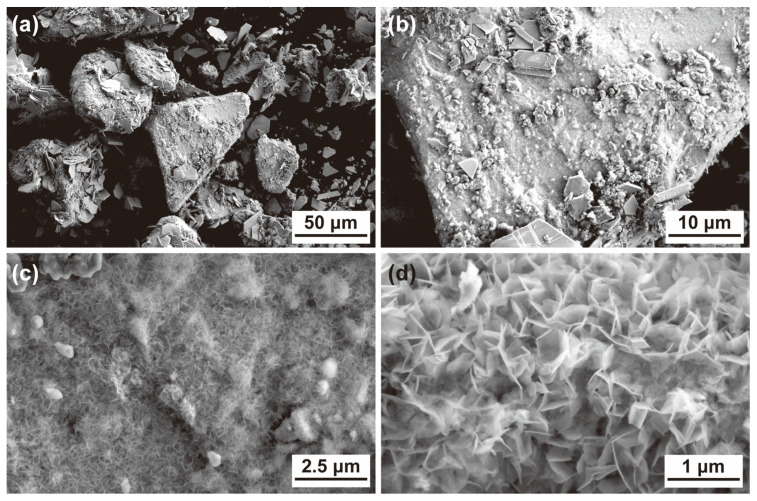
SEM images of the Zn_45_Mg_11_Ca_40_Sr_4_ alloy powder after degrading DB6 for 60 min at different magnifications: (**a**) 500×; (**b**) 2000×; (**c**) 10,000×; (**d**) 20,000×.

Figure 3 presents an SEM image of the Zn_45_Mg_11_Ca_40_Sr_4_ alloy powder after 60 min of degradation in the DB6 solution. The low-magnification SEM image in Figure 3a reveals sheet-like deposits with regular shapes on the particle surfaces, measuring approximately 20 μm in size, with most of them detaching from the alloy particles. Figure 3b, at 2000× magnification, displays not only the sheet-like deposits but also clusters of nearly spherical particles in the micrometer range, which are actually composed of smaller nanoparticles (Figure 3c). At 20,000× magnification, Figure 3d clearly shows that these nanoparticle clusters are agglomerations of dense nanosheets, which, in turn, form a submicron-scale porous network covering the entire matrix. This network structure, commonly observed on the metal material surfaces used for azo dye degradation, is a characteristic phenomenon that catalyzes the degradation of azo dyes [19,20].

**Figure 4:**
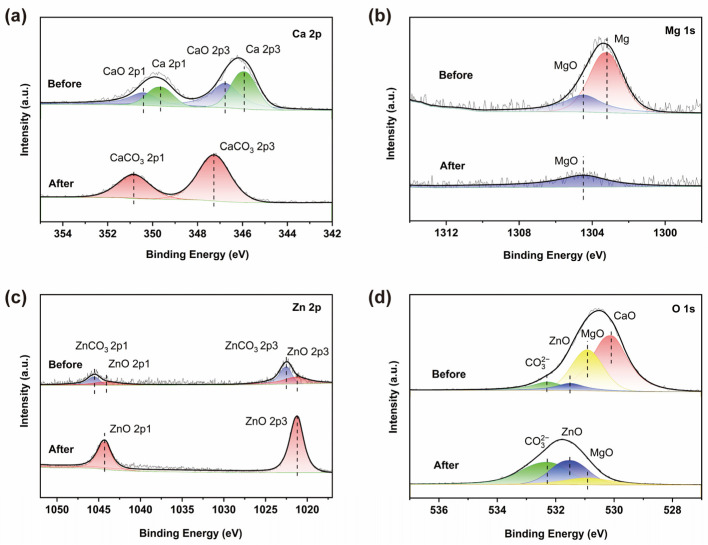
XPS spectra of the Zn_45_Mg_11_Ca_40_Sr_4_ alloy powder before and after azo dye degradation test: (**a**) Ca 2p; (**b**) Mg 1s; (**c**) Zn 2p; (**d**) O 1s.

Considering that the degradation reaction of the azo dyes is concentrated on the surface of the powder and there are fewer degradation products, XPS suitable for thin-layer samples was used to test the alloy powder before and after the azo dye degradation experiment. Figure 4a shows that the Ca element on the surface of the sample existed in the form of the zero-valent metal Ca and the oxide CaO before the reaction and was completely transformed into CaCO_3_ after the reaction. Figure 4b shows that the Mg element exists in the form of metallic Mg and MgO in the sample. After reacting with the azo dye solution, the zero-valent metal Mg is consumed in large quantities and disappears. On the contrary, the peak intensity of ZnO significantly increased after degradation by the azo dyes, as shown in Figure 4c, indicating that more Zn elements were exposed on the surface of the sample in the form of oxides. In addition, the changes in the binding energy of the O element in the samples before and after the degradation test of the azo dyes are shown in Figure 4d, which further proves that the Mg and Ca elements on the surface of the samples are greatly consumed during the reaction process, leading to an increase in the relative content of ZnO (with a lower Sr content and no corresponding information detected).

According to the results shown in Figure 3 and Figure 4, the main deposits on the surface of the alloy powder are Zn carbonate, hydroxide, and oxide. In fact, Zn can form a sheet-like Zn(OH)_2_ morphology in electrochemical deposition [21], which can be easily transformed into ZnO after drying. Therefore, this can indirectly prove that the nano-network in Figure 4 is a ZnO-like deposit.

As known, zero-valent metals can produce reducing [H] by reacting with water, which can destroy the azo groups in azo dyes. The Zn_45_Mg_11_Ca_40_Sr_4_ alloy we designed may react in the following order:

(1) $$Me^{0}\to {Me}^{2+}+e^{-}\;Me=(Zn,Ca,Mg)$$

(2) $$H_{2}O\to OH^{-}+H^{+}$$

(3) $$H^{+}+e^{-}\to [H]$$

(4) $$4\left[H\right]+Ar[-N=N-]Ar\to 2Ar[-NH_{2}]$$

Through Equations (1)–(4), Zn, Ca, and Mg on the particle surface are oxidized, generating a large amount of [H], which reacts with the azo dye molecule Ar[-N=N-]Ar, thereby breaking the azo bond. Subsequently, divalent metal ions of Me^2+^ can react with -OH in aqueous solutions or with [CO_3_]^2−^ formed by the dissolution of CO_2_, leading to the formation of carbonates or hydroxides. Over time, hydroxides can transform into more stable oxides, such as ZnO, which has photocatalytic properties. When ZnO is activated by visible light, hydroxyl radicals (•OH) and superoxide radicals (•O^2−^) can be produced, which can further oxidize and decompose azo dyes [22]. In addition, the aromatic by-products (Ar[-NH_2_]) resulting from the aforementioned reaction continue to pose a pollution risk, yet aromatic pollutants are comparatively easier to degrade than azo dyes due to the absence of stable -N=N- double bonds. Utilizing the identical oxidation–reduction and photocatalytic mechanisms, these aromatic compounds can be further degraded into shorter molecules, ultimately transforming into H_2_O, CO_2_, and harmless inorganic salts.

In the present work, we utilized non-toxic Zn, Ca, Mg, and Sr as the primary components to develop a new metallic glass composite material capable of rapidly degrading azo dyes. Based on the premise of the same powder particle size, the Zn-Ca-based metallic glass composite material developed in this work has a faster degradation rate and a lower activation energy than alloys such as Mg-Zn-Ca [14] and other metallic glass alloys. In addition, this wastewater treatment material, predominantly composed of lightweight elements, effectively mitigates the secondary risks associated with heavy metal ions, marking an environmentally friendly innovation with immense potential applications.

## 4. Conclusions

The Zn_45_Mg_11_Ca_40_Sr_4_ alloy powder, designed primarily with non-toxic lightweight elements, was successfully prepared through melt spinning and mechanical grinding. XRD and DSC analyses indicate that the powder material contains a large amount of the metallic glass phase and the CaZn_2_ crystalline phase, making it a typical in situ metallic glass composite material. This alloy powder can rapidly degrade Direct Blue 6 solution within the temperature range of 0–60 °C and can remove ~77% of the azo dye molecules within 1 min. The kinetic experiments and calculation results indicate that the degradation of azo dyes by the alloy powder is a thermal activation process, with a reaction activation energy of 8.99 kJ/mol. After the degradation reaction, a nanosheet-like sediment network was formed on the surface of the alloy, exhibiting the typical surface morphology characteristics of a zero-valent metal in degrading azo dyes, indicating that the azo dye degradation behavior of the alloy powder mainly follows the zero-valent metal reduction mechanism. This work provides a new perspective on the development of rapid degradation technology for azo dyes.

## Data Availability

The original contributions presented in the study are included in the article, further inquiries can be directed to the corresponding author.
